# Development and Validation of Computerized Adaptive Assessment Tools for the Measurement of Posttraumatic Stress Disorder Among US Military Veterans

**DOI:** 10.1001/jamanetworkopen.2021.15707

**Published:** 2021-07-08

**Authors:** Lisa A. Brenner, Lisa M. Betthauser, Molly Penzenik, Anne Germain, Jin Jun Li, Ishanu Chattopadhyay, Ellen Frank, David J. Kupfer, Robert D. Gibbons

**Affiliations:** 1 VA Rocky Mountain Mental Illness Research, Education and Clinical Center, Rocky Mountain Regional Veterans Affairs Medical Center, Eastern Colorado Health Care System, Aurora; 2 Department of Physical Medicine & Rehabilitation, University of Colorado, Anschutz Medical Campus, Aurora; 3 Department of Psychiatry & Neurology, University of Colorado, Anschutz Medical Campus, Aurora; 4 Pittsburgh School of Medicine, Pittsburgh, Pennsylvania; 5 Department of Medicine, University of Chicago, Chicago, Illinois; 6 Department of Computer Science, University of Chicago, Chicago, Illinois; 7 Committee on Quantitative Methods, University of Chicago, Chicago, Illinois; 8 Committee on Genetics, Genomics & Systems Biology, University of Chicago, Chicago, Illinois; 9 Center for Health Statistics, University of Chicago, Chicago, Illinois

## Abstract

**Importance:**

Veterans from recent and past conflicts have high rates of posttraumatic stress disorder (PTSD). Adaptive testing strategies can increase accuracy of diagnostic screening and symptom severity measurement while decreasing patient and clinician burden.

**Objective:**

To develop and validate a computerized adaptive diagnostic (CAD) screener and computerized adaptive test (CAT) for PTSD symptom severity.

**Design, Setting, and Participants:**

A diagnostic study of measure development and validation was conducted at a Veterans Health Administration facility. A total of 713 US military veterans were included. The study was conducted from April 25, 2017, to November 10, 2019.

**Main Outcomes and Measures:**

The participants completed a PTSD-symptom questionnaire from the item bank and provided responses on the PTSD Checklist for *Diagnostic and Statistical Manual of Mental Disorders, Fifth Edition* (*DSM-5*) (PCL-5). A subsample of 304 participants were interviewed using the Clinician-Administered Scale for PTSD for *DSM-5*.

**Results:**

Of the 713 participants, 585 were men; mean (SD) age was 52.8 (15.0) years. The CAD-PTSD reproduced the Clinician-Administered Scale for PTSD for *DSM-5* PTSD diagnosis with high sensitivity and specificity as evidenced by an area under the curve of 0.91 (95% CI, 0.87-0.95). The CAT-PTSD demonstrated convergent validity with the PCL-5 (*r* = 0.88) and also tracked PTSD diagnosis (area under the curve = 0.85; 95% CI, 0.79-0.89). The CAT-PTSD reproduced the final 203-item bank score with a correlation of *r* = 0.95 with a mean of only 10 adaptively administered items, a 95% reduction in patient burden.

**Conclusions and Relevance:**

Using a maximum of only 6 items, the CAD-PTSD developed in this study was shown to have excellent diagnostic screening accuracy. Similarly, using a mean of 10 items, the CAT-PTSD provided valid severity ratings with excellent convergent validity with an extant scale containing twice the number of items. The 10-item CAT-PTSD also outperformed the 20-item PCL-5 in terms of diagnostic accuracy. The results suggest that scalable, valid, and rapid PTSD diagnostic screening and severity measurement are possible.

## Key Points

**Question:**

Can rapid psychometrically sound adaptive diagnostic screening and dimensional severity measures be developed for posttraumatic stress disorder?

**Findings:**

In this diagnostic study including 713 US military veterans, the Computerized Adaptive Diagnostic–Posttraumatic Stress Disorder measure was shown to have excellent diagnostic accuracy. The Computerized Adaptive Test–Posttraumatic Stress Disorder also provided valid severity ratings and demonstrated convergent validity with the Post-Traumatic Stress Disorder checklist for *Diagnostic and Statistical Manual of Mental Disorders, Fifth Edition*.

**Meaning:**

In this study, the Computerized Adaptive Diagnostic–Posttraumatic Stress Disorder and Computerized Adaptive Test–Posttraumatic Stress Disorder measures appeared to provide valid screening diagnoses and severity scores, with substantial reductions in patient and clinician burden.

## Teaser

This diagnostic study develops and validates a tool for use in diagnostic screening of US military veterans for posttraumatic stress disorder.

## Introduction

Posttraumatic stress disorder (PTSD) in US military veterans is recognized as one of the signature injuries of the conflicts in Iraq and Afghanistan. Fulton et al^1^ conducted a meta-analysis of 33 studies published between 2007 and 2013, and PTSD prevalence among Operations Enduring Freedom and Iraqi Freedom veterans was estimated at 23%. Disease burden associated with PTSD is also notable among veterans from previous conflicts. Magruder and colleagues^2^ estimated the temporal course of PTSD among Vietnam veterans and identified 5 mutually exclusive groups (ie, no PTSD, early recovery, late recovery, late onset, and chronic). Based on these findings, the authors suggested that PTSD remains “a prominent issue” for many who served.^2^^(p2)^ Among adults in the US without a history of military service, lifetime incidence of PTSD is estimated at 6.8%,^3^ with women being twice as likely as men to be diagnosed with the condition.^3,4^

Provision of evidence-based treatment for those with PTSD is contingent on accurate identification. Traditionally, this identification has required the use of measures developed using classical test theory (ie, summing responses to a fixed set of items).^5^ Limitations of classical test theory are amplified when measuring complex conditions, such as PTSD.^5^

Diagnostically, criterion A events of PTSD include “exposure to actual or threatened death, serious injury, or sexual violence.”^6^^(p271)^ Such exposure can be secondary to directly experiencing, witnessing, learning about (occurred in a close family member or friend), and/or experiencing repeated or extreme exposure to aversive details regarding 1 or more traumatic events. Symptom-based criteria include intrusive symptoms (eg, distressing memories of the events), avoidance of stimuli (eg, people and/or places that remind the affected person of the events), and negative alterations in cognitions and mood associated with the events (eg, feeling detached from others).^6^

As would be expected based on the above-stated criteria, individuals with PTSD experience a wide range of symptoms with varying severity. Using latent profile analysis, Jongedijk et al^7^ identified 3 classes of individuals among Dutch veterans with PTSD, including average, severe, and highly severe symptom severity classes. Among trauma-exposed, inner-city primary care patients, Rahman et al^8^ examined data to assess associations between PTSD subclasses and major depressive disorder. The investigators identified 4 subclasses, including high severity and comorbidity, moderate severity, low PTSD and high depression, and resilient. These findings highlight the need to identify strategies capable of measuring complex traits.

One alternative to administering traditional assessment measures is computerized adaptive testing (CAT) in which a person’s initial item responses are used to determine a provisional estimate of their standing on the measured trait, which is then used for the selection of subsequent items,^9^ thereby increasing the precision of measurement and accuracy of diagnostic screening and minimizing clinician and patient burden.^10^ For complex disorders, such as PTSD, in which items are selected from distinct yet related subdomains (eg, exposure, negative alteration in mood and/or cognition, alteration in arousal and/or activity, avoidance, and intrusion), selection of items is based on multidimensional rather than unidimensional item response theory (IRT).^11^ Adaptive diagnosis and measurement are fundamentally different. In measurement (ie, CAT) the objective is to move the items to the severity level of the patient. In computerized adaptive diagnosis (CAD), we move the items at the tipping point between a positive and negative diagnosis.^12^ Both methods are adaptive but are based on different statistical approaches. The CAT is based on unidimensional or multidimensional IRT and the model does not include an external criterion, such as a structured clinical interview (eg, Clinician-Administered PTSD Scale for the *Diagnostic and Statistical Manual of Mental Disorders, Fifth Edition* [*DSM-5*]^13^ [CAPS-5^14^]). External criteria, such as the CAPS-5, or extant measures, such as the PTSD Checklist for *DSM-5* (PCL-5^15^), can be used to validate the CAT, but these tools are not used to derive a CAT. By contrast, CAD is based on machine-learning models for supervised learning (eg, random forest). We can use the same set of symptom items as the CAT to derive a CAD, but here we need an external criterion, such as the CAPS-5, to train the machine-learning model. CAD adaptively derives a binary screening diagnosis with an associated level of confidence, and CAT derives a dimensional severity measure that can be used to assess the severity of the underlying disorder and change in severity over time. CAD and CAT are complementary but are fundamentally different in theory and application. To do large-scale screening and measurement of PTSD, both measures are needed.

Evidence for other mental health conditions (ie, depression,^16^ anxiety,^17^ mania/hypomania,^18^ psychosis,^19^ suicide risk,^20^ and substance use disorders^21^) indicates that one can create large item banks (hundreds of items for a given disorder), from which a small optimal subset of items can be adaptively administered for a given individual with no or minimal loss of information, yielding a substantial reduction in patient and clinician burden while maintaining high sensitivity and specificity for diagnostic categorization, as well as high correlation with extant self- and clinician-rated symptom severity standard measures. For CAD, Gibbons et al^12^ noted that the CAD for diagnosis of major depressive disorder reproduced the hour-long Structured Clinical Interview for *DSM-5* Research Version (SCID)^13^ diagnosis of major depressive disorder in less than a minute, using an average of 4 adaptively selected self-reported items, while maintaining sensitivity of 0.95 and specificity of 0.87 for the clinical *DSM-5* diagnosis. Such assessment tools (diagnostic screening [CAD] and severity assessment [CAT]) are currently lacking for PTSD based on *DSM-5* criteria.

Using *DSM-IV* criteria,^22^ Del Vecchio et al^23^ and Eisen et al^24^ developed an item bank and a CAT for PTSD using multidimensional IRT. Initially, the investigators conducted a systematic review of PTSD instruments to identify items representing each of the 3 symptom clusters (reexperiencing, avoidance, and hypervigilance), as well as 3 additional subdomains (depersonalization, guilt, and sexual problems). A 104-item bank was constructed. Eighty-nine of these items were retained to further develop and validate a computerized test for PTSD (P-CAT). Although the *DSM-5* was not completed at that time, the authors indicated that they included items related to domains that they expected to be included. Similarly, because *DSM-5* measures were not yet developed, validation measures (eg, civilian version of the PTSD Checklist)^25^ were based on *DSM-IV* criteria. Moreover, to “minimize burden and distress for participants,”^24^^(p118)^ the SCID PTSD module^26^ vs the Clinician-Administered PTSD scale^27^ was administered. Work by Weathers et al^28^ suggests that the CAPS is the most valid measure of PTSD relative to other clinical interviews or self-report measures. According to Eisen et al,^24^ although concurrent validity was supported by high correlations, sensitivity and specificity were variable and the P-CAT was found to not be as reliable among those with “low levels of PTSD.”^24^^(p1120)^ Although there are similarities between the CAT-PTSD and the P-CAT in terms of the underlying method, there are important differences as well. First, unlike the CAT-PTSD, which varies in length and has fixed precision of measurement, the P-CAT is fixed in length and allows the precision of measurement to vary. This difference has implications for longitudinal assessments in which constant precision of measurement is important and is assumed in most statistical models for the analysis of longitudinal data.^29^ Second, the P-CAT item bank was limited to 89 items, whereas our item bank has 211 items. As such, these new methods provide better coverage of the entire PTSD continuum and have more exchangeable items at any point on that continuum. Third, we have developed both a CAT for the measurement of severity and a CAD for diagnostic screening. Diagnostic screening based on a CAD generally outperforms thresholding a continuous CAT-based measure, using fewer items.^12^ The limitation of CAD is that it does not provide a quantitative determination, a gap that is filled by the CAT-PTSD. In combination, however, CAT and CAD can be used for both screening and measurement.

Based on *DSM-5* criteria, this study aimed to develop and test the psychometric properties of the CAD-PTSD (diagnostic screener) and the CAT-PTSD (dimensional severity measure) against the standard criterion measure (CAPS-5),^14^ as well as the PCL-5.^15^

## Methods

### Measure Development

We developed the CAD-PTSD and CAT-PTSD scales using the general method introduced by Gibbons and colleagues.^16^ First, a large item bank containing 211 PTSD symptom items was developed to create both the CAD-PTSD and CAT-PTSD measures, using separate analyses.

The CAT-PTSD measure was developed by first calibrating the item bank using a multidimensional IRT model (the bifactor model^30^) and then simulating CAT from the complete item response patterns (211 items) to select optimal CAT tuning parameters from 1200 different simulations. Next, the CAT-PTSD scale was validated against an extant PTSD scale, the PCL-5 (convergent validity) and the CAPS-5 (diagnostic discriminant validity). For CAD, we used an extremely randomized trees algorithm^31^ to develop a classifier for the CAPS-5 PTSD diagnosis based on adaptive administration of no more than 6 items from the bank.^12^ Classification accuracy was assessed using data not used to calibrate the model.

Most applications of IRT are based on unidimensional models that assume that all of the association between the items is explained by a single primary latent dimension or factor (eg, mathematical ability). However, mental health constructs are inherently multidimensional; for example, in the area of depression, items may be sampled from the mood, cognition, behavior, and somatic subdomains, which produce residual associations between items within the subdomains that are not accounted for by the primary dimension. If we attempt to fit such data to a traditional unidimensional IRT model, we will typically have to discard most candidate items to achieve a reasonable fit of the model to the data. Bock and Aitkin^32^ developed the first multidimensional IRT model, where each item can load on each subdomain that the test is designed to measure. This model is a form of exploratory item factor analysis and can accommodate the complexity of mental health constructs such as PTSD. In some cases, however, the multidimensionality is produced by the sampling of items from unique subdomains (eg, negative alterations in mood and/or cognition, avoidance, and intrusion). In such cases, the bifactor model, originally developed by Gibbons and Hedeker^33^ for binary response data and later extended by Gibbons et al^30^ for ordinal (polytomous) response data, permits each item to tap the primary dimension of interest (eg, PTSD) and 1 subdomain (eg, alterations in arousal and/or reactivity), thereby accommodating the residual dependence and allowing for the retention of most items in the final model. The bifactor model of Gibbons and Hedeker^33^ was the first example of a confirmatory item factor analysis model, and they suggested that it is computationally tractable regardless of the number of dimensions, in stark contrast to exploratory item factor analytic models. Furthermore, the estimated bifactor loadings are rotationally invariant, greatly simplifying interpretability of the model estimates. The bifactor model provides a parameter related to each item’s ability to discriminate high and low levels of the underlying primary and secondary latent variables, and severity parameters for the k-1 thresholds between the k ordinal response categories. The bifactor model produces a score and uncertainty estimate on the primary dimension for each participant as well as for each of the subdomains. Complete details regarding the models and estimation are provided by Gibbons and Hedeker^33^ for binary response data and Gibbons et al^30^ for ordinal response data.

Once the entire bank (ie, 211 PTSD items) is calibrated, we have estimates of each item’s associated severity and we can adaptively match the severity of the items to the severity of the person. We do not know the severity of the person in advance of testing, but we learn it as we adaptively administer items. Beginning with an item in the middle of the severity distribution, we administer the item, obtain a categorical response, estimate the person’s severity level and the uncertainty in that estimate, and select the next maximally informative item.^16^ This process continues until the uncertainty falls below a predefined threshold, in our case, 5 points on a 100-point scale. The CAT has several tuning parameters^16^ that we select by simulating CAT from the complete response patterns. Twelve hundred simulations are conducted, and we select the tuning parameters that minimize the number of items administered and maximize the correlation with the total bank score. The tuning parameters include the level of uncertainty at which we stop the adaptive test, a second stopping rule based on available information remaining in the item bank at the current level of severity, and an additional random component that selects the maximally informative item or the second maximally informative item to increase variety in the items administered. We select the next maximally informative item based on the following item information criteria. Item information describes the information contained in a given item for a specific severity estimate. Our goal is to administer the item with maximum item information at each step in the adaptive process.

Unlike a CAT, which is criterion-free, a CAD uses the diagnostic information (ie, external criterion) to derive a classifier based on a subset of the symptoms in the item bank that maximize the association between the items and the diagnosis. A CAD is used for diagnostic screening, whereas a CAT is used for symptom severity measurement. Gibbons et al^12^ developed the first CAD for major depressive disorder. CADs are based on machine learning methods, such as a random forest,^34^ of lower dimension than the dimensionality of the entire item bank, with the goal of minimizing loss of information from the full-bank classifier so that patient and clinician burden are minimized. The critical machine learning problem is to place a constraint on the number of features (symptom items) that may be used per sample. To this end, we used the extra-trees method (ie, extremely randomized trees)^31^ with the objective of further randomizing tree building in the context of numerical input features, where the choice of the optimal cutpoint is responsible for a large proportion of the variance of the induced tree. The choice of the extra-trees classifier was optimized among a set of possible state-of-the-art algorithms, including random forests, gradient descent, support vector machines, neural networks, and AdaBoost. The extra-trees approach produced superior out-of-sample classification performance, particularly under the restriction of the total number of items that may be used to reach a decision. In our model, we used 2 decision trees in each ensemble, where the depth of each tree is limited to 3, resulting in a total of 6 items being adaptively administered in each test. In addition, the use of 2 decision trees that are combined linearly allows for an additional degree of freedom over using a single decision tree depth of 6 (as used by Gibbons et al^12^), resulting in a substantial increase in performance. The large size of the item bank implies that several alternative models (all with similar high out-of-sample performance) could be obtained, allowing for the generation of a number of possible tests with different sets of items being administered in each test. For each patient, we randomly select 1 such extra-trees model and administer the test, which presents 6 items adaptively as we traverse the 2-component decision trees based on the specific patient responses. An example of a 3-estimator ensemble (1 of 2 used in constructing a test) is shown in the eFigure in the Supplement. It is possible to have the same item appear twice in a given test form, which results in the imputation of the earlier item response and a reduction in the number of uniquely identified items from, for example, 6 to 5. The extra-trees model has recently been used to develop a CAD for psychosis.^35^

### Participants

Veterans were eligible if they were between the ages of 18 and 89 years and able to provide written informed consent. Participants (n = 713) were recruited from a mountain state metropolitan Veterans Affairs Medical Center between April 25, 2017, and November 10, 2019. Institutional review board approval was obtained from the Colorado Multiple Institutional Review Board. Participants received financial compensation.

A convenience sample (n = 304) was recruited via flyers posted at the local Veterans Affairs Medical Center and surrounding community-based outpatient clinics. The research team also distributed study information to mental health and primary care clinicians at the local Veterans Affairs Medical Center to facilitate recruitment. In addition, veterans who had participated in previous research with existing consent to be contacted for future studies were mailed study-specific flyers with study contact information. Once eligibility was determined by the study team, participants were invited to complete an in-person study visit wherein clinical interview measures, including the CAPS-5, were administered by trained research personnel (L.M.B.). Interview schedules were reviewed by licensed clinicians (L.A.B. and L.M.B.) for quality management. Self-report measures were completed on paper, and the PTSD item bank was administered using research electronic data capture (REDCap).^36^

To recruit the remaining participants (n = 409) required for all planned analyses (n = 713), efforts were made to identify, via the VA Corporate Data Warehouse, those with varying levels of psychiatric symptom burden based on past mental health treatment obtained. This population included veterans who received services through an inpatient or outpatient psychiatric unit, PTSD residential program, mental health clinic, or primary care clinic at the local Veterans Affairs Medical Center or community-based outpatient clinics since 2009. Duplicates with those already enrolled were removed. Letters of invitation were sent to 9350 potential participants, 5.1% expressed interest, and 409 (4.4%) completed the study procedures. These 409 participants completed the PCL-5 and PTSD item bank via REDCap.^36^

### Measures

We developed an item bank containing 211 PTSD items drawn from 16 existing self-report and clinician-administered PTSD scales (eTable in the Supplement) and newly created items. Existing items were reworded to make them appropriate for adaptive administration, self-report, and user-selectable time frames. Items were drawn from 5 subdomains: exposure (5 items), negative alterations in mood/cognition (58 items), alterations in arousal/reactivity (79 items), avoidance (18 items), and intrusion (51 items). Items were rated on 4- or 5-point Likert scales with categories of not at all, a little bit, moderately, quite a bit, very much, never, rarely, sometimes, and often.

The trauma/PTSD L Module of the SCID^13^ was used to assess criterion A events and the presence of symptoms. If a criterion A event and at least 1 current symptom were endorsed, the CAPS-5 was administered.^14^ The CAPS-5 is the standard for assessing PTSD diagnosis.^28^ Non-PTSD modules of the SCID^13^ were administered to obtain information regarding current mental health conditions. The PCL-5^15^ was used to determine self-reported PTSD symptom severity.

### Statistical Analysis

The bifactor IRT models were fitted with the POLYBIF program. Improvement in fit of the bifactor model over a unidimensional alternative was determined using a likelihood ratio χ^2^ statistic. The extra-trees classification algorithm was fitted using the Scikit-learn Python library. Logistic regression was used to estimate diagnostic discrimination capacity for the CAT-PTSD and area under the curve (AUC) for the receiver operating characteristic curve with 10-fold cross-validation using Stata, version 16 (StataCorp LLC). The Pearson *r* correlation coefficient test was used to assess the association between the CAT-PTSD score and the PCL-5 score. Using 2-sided testing, findings were considered significant at *P* < .05.

## Results

### Participants

In Table 1, demographic characteristics are provided for the study sample, as well as for the subsample that completed diagnostic interviews. Of the 713 participants, 585 (82.3%) were men and 126 (17.7%) were women; mean (SD) age was 52.8 (15.0) years. Clinical characteristics regarding the subsample are presented in Table 2.

**Table 1.** Sample Characteristics^a^

Characteristic	Full sample (N = 713)	Participants who completed diagnostic interviews (n = 304)
Age, y		
No. of respondents	713	304
Mean (SD)	52.8 (15.0)	47.1 (12.6)
Median (range)	54 (22-83)	47 (22-77)
Sex, No. (%)		
No. of respondents	711	304
Male	585 (82.3)	246 (80.9)
Female	126 (17.7)	58 (19.1)
Race, No. (%)		
No. of respondents	710	304
Caucasian or White	558 (78.6)	221 (72.7)
Black or African American	82 (11.6)	49 (16.1)
Native American/Alaskan Native	9 (1.3)	4 (1.3)
Asian	6 (0.9)	1 (0.3)
Pacific Islander	4 (0.6)	2 (0.7)
Multiracial	36 (5.1)	19 (6.3)
Other	15 (2.1)	8 (2.6)
Ethnicity, No. (%)		
No. of respondents	710	304
Hispanic or Latino/a	70 (9.9)	45 (14.8)
Not Hispanic or Latino/a	640 (90.1)	259 (85.2)
Educational level, No. (%)		
No. of respondents	712	303
9th-12th grade, no diploma	4 (0.6)	1 (0.3)
High school diploma or equivalent	65 (3.1)	45 (14.9)
Some college, no degree	172 (24.2)	83 (27.4)
Associate’s degree	110 (15.5)	48 (15.8)
Bachelor’s degree	181 (25.4)	64 (21.1)
Master’s degree	161 (22.6)	56 (18.5)
Doctoral degree	19 (2.7)	6 (2)
Marital status, No. (%)		
No. of respondents	712	304
Married	379 (53.2)	131 (43.1)
Single	133 (18.7)	79 (26.0)
Cohabitating	28 (3.9)	15 (4.9)
Widowed	16 (2.3)	4 (1.3)
Divorced/separated	156 (21.9)	75 (24.7)
Sexual orientation, No. (%)		
No. of respondents	709	304
Heterosexual/straight	662 (93.4)	279 (91.8)
Gay/lesbian	27 (3.8)	16 (5.3)
Bisexual	17 (2.4)	9 (2.9)
Questioning	2 (0.3)	0
Other	1 (0.1)	0
Employment status, No. (%)		
No. of respondents	706	302
Employed full-time	226 (32.0)	88 (29.1)
Employed part-time	74 (10.5)	31 (10.3)
Unemployed, not currently seeking employment	104 (14.7)	74 (24.5)
Unemployed, seeking employment	63 (8.9)	37 (12.3)
Retired	239 (33.9)	72 (23.8)
Student status, No. (%)		
No. of respondents	711	303
Currently a student	82 (11.5)	49 (16.2)
Not currently a student	629 (88.5)	254 (83.8)
Currently homeless, No. (%)		
No. of respondents	711	304
No	695 (97.7)	294 (96.7)
Yes	16 (2.3)	10 (3.3)
Unique episodes of homelessness, No.		
No. of respondents	711	302
Mean (SD)	0.7 (1.9)	1.2 (2.6)
Median (range)	0 (0-25)	0 (0-25)
Branch of military service, No. (%)		
No. of respondents	712	303
Army	390 (54.8)	179 (59.1)
Air Force	137 (19.2)	46 (15.2)
Navy	83 (11.7)	34 (11.2)
Marines	62 (8.7)	32 (10.6)
Coast Guard	3 (0.4)	0
Multiple branches	37 (5.2)	12 (3.9)
Highest rank, No. (%)		
No. of respondents	710	302
Enlisted	466 (65.6)	228 (75.5)
Noncommissioned officer	137 (19.3)	48 (15.9)
Warrant officer	8 (1.1)	1 (0.3)
Officer	99 (13.9)	25 (8.3)
Deployments, No.		
No. of respondents	713	304
Mean (SD)	2.1 (3.2)	2.1 (3.5)
Median (range)	1 (0-40)	1 (0-40)
Deployments to combat zone, No.		
No. of respondents	712	304
Mean (SD)	1.0 (1.5)	5.5 (1.8)
Median (range)	1 (0-20)	1 (0-20)
Years of active duty service, No.		
No. of respondents	699	304
Mean (SD)	8.2 (7.5)	7.2 (6.4)
Median (range)	4.5 (0-36.9)	4.4 (0-30)
Years of reserve service, No.		
No. of respondents	707	304
Mean (SD)	1.8 (4.2)	2.2 (4.7)
Median (range)	0 (0-27.6)	0 (0-27.5)

^a^ Some participants declined to respond to certain items; in these cases, the number who responded to that item or measure is reported.

**Table 2.** Clinical Characteristics of Participants Who Completed Diagnostic Interviews (n = 304)

Characteristic	No. (%)
PTSD criterion A (Structured Clinical Interview for *DSM-5* Research Version)	303
No. (%)	178 (58.8)
Current PTSD (Clinician-Administered PTSD Scale for *DSM-5*)	303
No. (%)	86 (28.4)
Current mental health conditions (Structured Clinical Interview for *DSM-5* Research Version), No. (%)	304
Current bipolar disorder (includes bipolar 1 and bipolar 2 disorder)	12 (3.9)
Current major depressive disorder	80 (26.3)
Current alcohol use disorder	27 (8.9)
Current substance use disorder	28 (9.2)
Current generalized anxiety disorder	11 (3.6)
Current sleep disorders (insomnia and hypersomnia)	21 (6.9)

Abbreviations: *DSM-5*, *Diagnostic and Statistical Manual of Mental Disorders, Fifth Edition*; PTSD, posttraumatic stress disorder.

Data from 713 participants were used to calibrate the 211 PTSD items. Following removal of 8 items with poor discrimination (loadings <0.3) on the primary dimension, 203 items remained (final item bank). The bifactor model significantly improved the fit over a unidimensional IRT alternative (χ^2^ = 14 928_203_; *P* < .001).

### CAT-PTSD and CAD-PTSD Development

Simulated adaptive testing from complete PTSD item bank responses revealed that using a mean of 10 items per participant (range, 4-17) in the CAT-PTSD, we maintained a correlation of *r* = 0.95 with the 203-item total bank score (a 95% reduction). Median length of assessment was 59 seconds (interquartile range, 32-117 seconds).

To aid in patient triage, severity thresholds were selected based on sensitivity and specificity for the CAPS-5 diagnosis of PTSD. Scores on the CAT-PTSD can range from 0 to 100 and map on to PTSD severity categories. Categories of none, mild, moderate, and severe were selected; the shift between none and mild was selected to have high sensitivity and moderate specificity, mild vs moderate to have high sensitivity and high specificity, and moderate vs severe to have high specificity.^16^ These decision rules yielded thresholds of CAT-PTSD scores of 35 (95% sensitivity, 51% specificity) for none vs mild, 47 (79% sensitivity, 78% specificity) for mild vs moderate, and 60 (50% sensitivity, 93% specificity) for moderate vs severe. Although the actual score should be relied on for measurement and the assessment of change, these categories may be useful for clinical decision-making.

Validation occurred using 304 individuals who participated in CAPS-5 diagnostic interviews and 713 who participated in the PCL-5. We validated the CAT-PTSD against the CAPS-5 diagnosis (diagnostic validity) and against the PCL-5 (convergent validity). Diagnostic predictive validity for the CAT-PTSD was demonstrated against the CAPS-5 (AUC = 0.85; 95% CI, 0.79-0.89) using 10-fold cross-validation so that classification accuracy was assessed in patients not used in estimating the logistic regression. This AUC represents excellent discrimination per Hosmer et al.^37^ No loss of diagnostic predictive accuracy between the full 203-item bank classification (AUC = 0.84; 95% CI, 0.79-0.89) and the 10-item (average) CAT-PTSD was found, demonstrating that the CAT extracted full information from the total item bank using a mean of only 10 items from the 203 items. By contrast, the diagnosis based on the PCL-5 (using a threshold score of 33) had lower diagnostic predictive accuracy (AUC = 0.75; 95% CI, 0.68-0.82) using twice the number of items (20 items). Convergent validity between the CAT-PTSD and PCL-5 was demonstrated (*r* = 0.88; *P* < .001).

Data from 304 participants with CAPS-5 diagnostic interviews were used to calibrate the CAD-PTSD, using all 211 items. The 6-item CAD-PTSD (a 97% reduction in items) produced a cross-validated AUC of 0.91 (95% CI, 0.87-0.95) for the clinician-rated CAPS-5 PTSD diagnosis (Figure). This level falls in the category of outstanding discrimination.^37^ Mean test time was 35 seconds (interquartile range, 19-70 seconds). Combined use of the CAD-PTSD and CAT-PTSD was a mean of 94 seconds.

**Figure.**
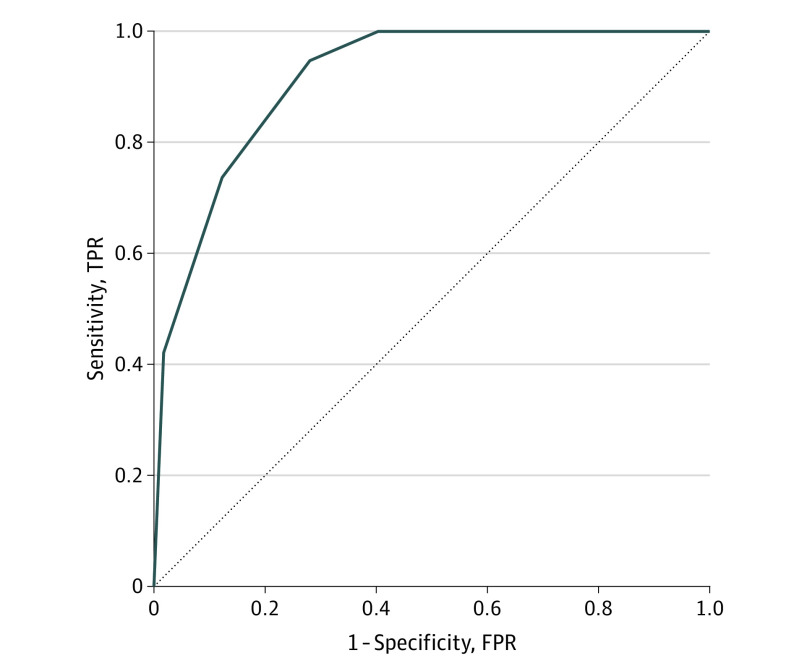
Receiver Operating Characteristic Curve for the Computerized Adaptive Diagnostic (CAD) Posttraumatic Stress Disorder (PTSD) Measure With the receiver operating characteristic curve for the prediction of the *Diagnostic and Statistical Manual of Mental Disorders, Fifth Edition* diagnosis of PTSD based on the CAD-PTSD classifier, the area under the curve was 0.91. FPR indicates false-positive rate; TPR, true-positive rate.

In Table 3, example CAT-PTSD interviews for patients with low, moderate, and high PTSD severity are presented. The testing session result is classification as having no evidence of PTSD (requires 12 items), possible PTSD (requires 9 items), and PTSD definite or highly likely (requires 11 items). In Table 4, examples of negative and positive CAD-PTSD diagnostic screening interviews are presented. The probability of PTSD is 0.01 (requires 6 items) for the negative interview and 0.81 (requires 5 items) for the positive interview.

**Table 3.** Example CAT-PTSD Sessions by Severity^a^

Severity level	Response	Score	Uncertainty
**Low: no evidence of PTSD**			
How much have you been bothered by having to avoid activities or situations because they reminded you of a stressful experience from the past?	Not at all	26.72	11.83
How much of a problem have you had with a loss of interest in your usual activities due to a stressful event in the past?	A little	29.65	8.91
How much were you bothered by feeling very upset when something reminded you of a stressful experience from the past?	A little	32.73	7.3
How much were you distressed or bothered by staying away from reminders of a stressful event in the past?	Not at all	31.01	7.13
How much were you distressed or bothered by trying not to think about a stressful event in the past?	Not at all	29.15	6.97
How much have you been bothered by repeated disturbing memories, thoughts, or images of a stressful experience from the past?	A little	29.59	6.76
How much did trying to avoid thoughts, feelings, or physical sensations that reminded you of a stressful experience occur or become worse?	Not at all	28.82	6.71
How much were you distressed or bothered by trouble staying asleep due to a stressful event in the past?	Not at all	25.38	6.38
How much of a problem have you had with restlessness due to a stressful event in the past?	A little	27.57	5.72
How much were you distressed or bothered by other things that kept making you think about a stressful event in the past?	Not at all	25.42	5.62
How much were you bothered by repeated, disturbing, and unwanted memories of a stressful experience from the past?	Not at all	24.47	5.58
How much of a problem have you had with irritability due to a stressful event in the past?	A little	25.92	5
**Moderate severity: possible PTSD**			
How much have you been bothered by having to avoid activities or situations because they reminded you of a stressful experience from the past?	Moderately	54.55	10.07
How much were you distressed or bothered by trying to remove a stressful event in the past from your memory?	Moderately	55.1	9.27
How much did trying to avoid thoughts, feelings, or physical sensations that reminded you of a stressful experience occur or become worse?	Moderately	54.59	8.96
How much have you been bothered by avoiding having to think about or talking about a stressful experience from the past or avoiding having feelings related to it?	Moderately	54.65	8.86
How much were you distressed or bothered by trying not to think about a stressful event in the past?	Moderately	54.87	8.64
How much were you bothered by repeated, disturbing, and unwanted memories of a stressful experience from the past?	A little bit	50.32	7.37
How much were you distressed or bothered by trouble staying asleep due to a stressful event in the past?	Moderately	50.11	6.67
How much did feeling very emotionally upset when something would remind you of a stressful experience, occur or become worse?	Quite a bit	55.13	6.31
How much were you distressed or bothered by being jumpy and easily startled due to a stressful event in the past?	Moderately	55.05	4.99
**High severity: PTSD definite or highly likely**			
How much have you been bothered by having to avoid activities or situations because they reminded you of a stressful experience from the past?	Quite a bit	63.56	10
How much were you distressed or bothered by trying to remove a stressful event in the past from your memory?	Very much	70.55	9.36
How much have you been bothered by suddenly acting or feeling as if a stressful experience were happening again?	Very much	80.08	7.94
How much were you distressed or bothered by trying not to think about a stressful event in the past?	Very much	82.66	7.78
How much have you been bothered by repeated disturbing memories, thoughts, or images of a stressful experience from the past?	Very much	81.91	7.2
How much were you distressed or bothered by thinking about a stressful event in the past when you didn't mean to?	Quite a bit	79.71	6.92
How much were you distressed or bothered by avoiding getting upset when you thought about a stressful event in the past or was reminded of it?	Very much	81.51	6.74
How much did feeling very emotionally upset when something would remind you of a stressful experience, occur or become worse?	Very much	82.78	6.66
How much were you bothered by avoiding memories, thoughts, or feelings related to a stressful experience from the past?	Very much	83.52	5.64
How much did trying to avoid thoughts, feelings, or physical sensations that reminded you of a stressful experience occur or become worse?	Very much	83.93	5

Abbreviations: CAT, computerized adaptive test; PTSD, posttraumatic stress disorder.

^a^ The testing session result is classified as no evidence of PTSD (requires 12 items), possible PTSD (9 items), and PTSD definite or highly likely (11 items).

**Table 4.** Example Negative and Positive CAD-PTSD Sessions^a^

Item	Response
**Interview 1: negative**^**b**^	
I tried not to think about things that remind me of something bad that happened to me	Rarely
My daydreams were very real and frightening	A little bit
How much were you bothered by suddenly feeling or acting as if a stressful experience from the past was actually happening again (as if you were actually back there reliving it)?	Not at all
Have you felt on edge, been easily distracted, or had to stay “on guard”?	A little bit
I got very upset when something reminded me of something bad that happened to me	Never
How much were you distressed or bothered by feeling as if a stressful event in the past hadn’t happened or wasn’t real?^c^	Not at all
Diagnosis: negative	Probability of having PTSD *P* = .01
**Interview 2: positive**^**b**^	
How much were you bothered by repeated, disturbing dreams of a stressful experience from the past?	Very much
How much did feelings of being “super alert,” on guard, or constantly on the lookout for danger occur or become worse after a stressful event or experience in the past?^c^	Very much
Have you markedly lost interest in free-time activities that used to be important to you?	Often
How much did having a very negative emotional state occur or become worse after having a stressful event or experience?	Very much
Someone touched me in a sexual way against my will^d^	Often
Diagnosis: positive	Probability of having PTSD *P* = .81

Abbreviations: CAD, computerized adaptive diagnostic; PTSD, posttraumatic stress disorder.

^a^ The probability of PTSD is 0.01 (requires 6 items) for the negative interview and 0.81 (5 items) for the positive interview.

^b^ Items begin with “In the past month.”

^c^ Item stem changes to “During the past month.”

^d^ Item stem changes to “During a stressful event in the past.”

## Discussion

Among US military veterans, we have developed an adaptive diagnostic screener and a dimensional severity measure for PTSD and have examined their validity in terms of tracking results of a structured clinical interview and an extant severity measure. The CAD reproduced the CAPS-5 with outstanding diagnostic accuracy (AUC = 0.91). As expected, the CAD-PTSD outperformed the CAT-PTSD in terms of diagnostic accuracy (AUC = 0.91 vs AUC = 0.85), with a 40% reduction in the number of items (6 vs 10). Nevertheless, the CAT-PTSD had classification accuracy in the excellent range. The CAT-PTSD demonstrated convergent validity against the PCL-5 with high correlation (*r* = 0.88). Despite the high correlation, the CAT-PTSD outperformed the PCL-5 in terms of diagnostic accuracy (AUC = 0.85 vs 0.75), using half the number of items. Relative to the full item bank of 203 items, the CAD-PTSD had a 97% reduction of items and the CAT-PTSD had a 95% reduction of items while maintaining a correlation of *r* = 0.95 with the total 203-item bank score for the CAT-PTSD.

Integration of these adaptive tests within electronic health record systems^38^ can facilitate routine diagnostic screening and symptom severity measurement for PTSD in usual clinical care. In many cases, to further reduce burden, conditional testing can be used in which the CAD-PTSD is administered first as a diagnostic screener and the CAT-PTSD is administered only for patients who have positive screening results and require further characterization of their PTSD severity and categorization within clinically meaningful thresholds of mild, moderate, and severe. This will reduce median administration time to 35 seconds for most people, and 94 seconds for those with positive screening results. The CAD-PTSD item responses can be used to initiate the CAT-PTSD because the items are derived from the same 203-item bank, providing a further reduction in administration time.

The advantages of self-assessments over rater-based evaluations are substantial. Self-assessments are not limited to the availability of highly trained interviewers and can be administered in or out of the clinic on internet-capable devices (eg, smartphones). Self-assessments also eliminate interviewer bias and reduce costs, thereby enhancing scalability. During clinical visits, clinicians and patients can use saved time to focus on treatment planning. An additional advantage of adaptive tests is for longitudinal assessments in which response bias associated with repeated administration of the same items is eliminated because the items change across assessments. One might hypothesize that changing the items results in decreased test-retest reliability, but in fact the reverse is true. Beiser and colleagues^39^ showed that test-retest reliability was 0.92 for the adaptive depression test (the CAT-Depression Inventory)^16^ but only 0.84 for the fixed-length Patient Health Questionnaire-9.^40^

The CAT-PTSD produces a continuous severity score on a scale of 0 to 100 with 5 points of precision. The continuous severity score can be thresholded into clinically useful categories of none, mild, moderate, and severe, as we have illustrated. The continuous severity score from the CAT-PTSD is ideal for longitudinal assessments, because its precision is fixed on repeated measurements, in contrast to traditional short-form tests. Furthermore, the CAT-PTSD adapts to changing psychopathologic characteristics by targeting the severity of the items to the severity of the patient, further improving the precision of measurement and the ability to assess change. A recent ketamine randomized clinical trial^41^ found that CAT depression and suicidality measures, developed using the same technology, outperformed traditional fixed-length clinician-rated (Hamilton Depression Rating Scale^42^ and Beck Scale for Suicide Ideation^43^) and self-rated (Beck Depression Inventory^44^) measures in terms of sensitivity to change.

Future directions include the need for additional field testing, which would also allow for evaluation of the acceptability and feasibility of implementing these tools in clinical settings, including via telehealth, which has been increasingly implemented as a result of the COVID-19 pandemic. Use of telehealth assessment will in part be facilitated by designing a graphical user interface^45^ in a cloud computing environment for routine test administration on internet-capable devices, such as smartphones, tablets, notebooks, and computers, and providing an advanced programming interface that can be interfaced with the electronic health record. To accommodate literacy issues, audio to the self-report questions can be enabled. Because the generation and testing of subdomain scores is beyond the scope of this study, future research in this area is warranted.

### Limitations

This study has limitations. The CAD-PTSD and CAT-PTSD do not allow for evaluation and monitoring of specific symptoms to the extent that they may not always be adaptively administered. However, items from the 5 subdomains are available from most interviews and can be used to assess specific subdomains of PTSD (eg, avoidance). In addition, this study was conducted exclusively in English. Independent replication of our findings in other patient populations and in other languages (eg, Spanish) is needed.^46^

## Conclusions

The findings of this study suggest that, among veterans, the CAD-PTSD and CAT-PTSD appear to provide valid diagnostic screening and dimensional severity scores, with substantial reductions in patient and clinician burden. These measures are scalable and can be integrated into electronic health record systems for routine use in health care settings.

## Notes

Supplement. **eTable.** Existing Scales From Which the Item Bank Was Constructed **eFigure.** One of Two Decision Trees for a Test in the CAD-PTSD (Exemplar)
