# Supplementary material for: Development and Validation of Computerized Adaptive Assessment Tools for the Measurement of Posttraumatic Stress Disorder Among US Military Veterans
Source: JAMA Netw Open. 2021 Jul 8;4(7):e2115707. doi: 10.1001/jamanetworkopen.2021.15707 (PMC8267606; doi:10.1001/jamanetworkopen.2021.15707)
Supplement: Supplement. — eTable. Existing Scales From Which the Item Bank Was Constructed eFigure. One of Two Decision Trees for a Test in the CAD-PTSD (Exemplar) [file jamanetwopen-e2115707-s001.pdf]

Supplementary Online Content

Brenner LA, Betthauser LM, Penzenik M, et al. Development and validation of computerized adaptive assessment tools for the measurement of posttraumatic stress disorder among US military veterans. *JAMA Netw Open*. 2021;4(7):e2115707. doi:10.1001/jamanetworkopen.2021.15707

eTable. Existing Scales From Which the Item Bank Was Constructed

eFigure. One of Two Decision Trees for a Test in the CAD-PTSD (Exemplar)

This supplementary material has been provided by the authors to give readers additional information about their work.

© 2021 Brenner LA et al. *JAMA Network Open.*

eTable. Existing Scales from which the Item Bank Was Constructed

Measure	Abbreviation	Citation
Combat Exposure Scale	CES	Keane T, Fairbank J, Caddell J, Zimering R, Taylor K, Mora C. Clinical evaluation of a measure to assess combat exposure. *Psychological Assessment.* 1989;1(1):53-55
Davidson Trauma Scale	DTS	Davidson J. Davidson Trauma Scale (DTS). North Tonawanda, NY: Multi-Health Systems, Inc. 1996
Deployment Risk and Resilience Inventory-2	DRRI-2	Vogt D, Smith BN, King LA, King DW, Knight J, Vasterling JJ. Deployment Risk and Resilience Inventory-2 (DRRI-2): An updated tool for assessing psychosocial risk and resilience factors among service members and veterans. *Journal of Traumatic Stress*. 2013;26(6):710-717. doi: 10.1002.jts.21868 PILOTS ID: 87988
Impact of Event Scale - Revised	IES-R	Weiss DS, Marmar CR. The Impact of Event Scale-Revised. In J.P. Wilson, & T.M. Keane (Eds.), Assessing Psychological Trauma and PTSD: A Practitioner's Handbook. New York: Guilford Press. 1997;399-411
Los Angeles Symptoms Checklist	LASC	King LA, King DW, Leskin GA, Foy DW. (1995). The Los Angeles Symptom Checklist: a self-report measure of posttraumatic stress disorder. *Assessment.* 1995;2:1-17
Minnesota Multiphasic Personality Inventory 2 PK scale	MMPI-2, PK Scale	Lyons JA, Terence MK. "Keane PTSD scale: MMPI and MMPI-2 update." *Journal of Traumatic Stress*. 1992;5(1):111-117
Modified PTSD Symptom Scale	MPSS-SR	Falsetti SA, Resnick HS, Resick PA, Kilpatrick DG. The Modified PTSD Symptom Scale: A brief self-report measure of posttraumatic stress disorder. *The Behavior Therapist.* 1993;16(6):161-162
National Stressful Events Survey Acute Stress Disorder Short Scale - Severity of PTS Symptoms	NSESSS	Kilpatrick DG, Resnick HS, Friedman MJ. National Stressful Events Survey PTSD Short Scale (NSESSS-PTSD*). Scale available from* *http://www.psychiatry.org/practice/dsm/dsm5/online-assessment-measures#Disorder*. 2013

© 2021 Brenner LA et al. *JAMA Network Open.*

Personality Assessment Inventory, Traumatic Stress Subscale	PAI; ARD-T	Morey LC. The Personality Assessment Inventory professional manual. Odessa, FL: Psychological Assessment Resources. 1991
Primary Care PTSD Screen	PC-PTSD	Prins A, Ouimette P, Kimerling R, Cameron RP, Hugelshofer DS, Shaw-Hegwer J, Thrailkill A, Gusman FD, Sheikh JI. The Primary Care PTSD Screen (PC-PTSD). *Primary Care Psychiatry*. 2004;9:151
PTSD Check List - Civilian	PCL-C	Blanchard EB, Jones AJ, Buckley TC, Forneris CA. Psychometric properties of the PTSD Checklist (PCL). *Behaviour Research and Therapy*. 1996;34:669-673
PTSD Checklist 5	PCL-5	Weathers FW, Litz BT, Keane TM, Palmieri PA, Marx BP, Schnurr PP. The PTSD Checklist for DSM-5 (PCL-5). *Scale available from the National Center for PTSD at www.ptsd.va. gov.* 2013;10
Revised Civilian Mississippi Scale	RCMS	Norris FH, Perilla J. Reliability, validity, and cross-language stability of the Revised Civilian Mississippi Scale for PTSD. *Journal of Traumatic Stress.* 1996;9:285-298
Screen for Post Traumatic Stress Symptoms	SPTSS	Carlson E. The Screen for Posttraumatic Stress Symptoms (SPTSS) . Measurement Instrument Database for the Social Science. *Scale available from www.midss.org*. 2012
The Mississippi Scale for Combat-Related PTSD	M-PTSD	Keane TM, Caddell JM, Taylor KL. Mississippi scale for combat-related posttraumatic stress disorder: Three studies in reliability and validity. *Journal of Consulting and Clinical Psychology*. 1988;56:85-90
Trauma Symptom Checklist 40	TSC-40	Briere J, Runtz M. The Trauma Symptom Checklist (TSC-33): Early data on a new scale. *Journal of Interpersonal Violence.* 1989;4:151-163

© 2021 Brenner LA et al. *JAMA Network Open.*

eFigure. One of Two Decision Trees for a Test in the CAD-PTSD (Exemplar)

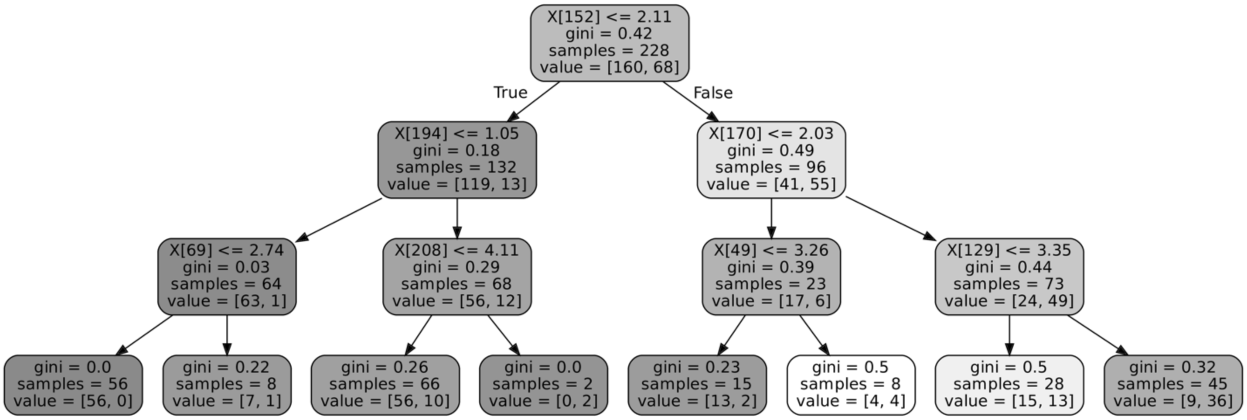

© 2021 Brenner LA et al. *JAMA Network Open.*
